# 

**Published:** 1906-02

**Authors:** 


The Conceit of the Reformers.

VOL. XXI. FEBRUARY, 1906. No. 8.

LIBRARY INDEXED [illegible]

### TEXAS Medical Journal.

SUBSCRIPTION, $1.00 A YEAR, IN ADVANCE.

SINGLE COPIES, 10 CENTS.

PUBLISHED AT AUSTIN, TEXAS, BY
F. E. DANIEL, M. D.,
PROPRIETOR.

PRINTED BY THE VON BOECKMANN-JONES CO.

Entered at the Postoffice at Austin, Texas, as Second-class Mail Matter.

